# The Application of Multiobjective Genetic Algorithm to the Parameter Optimization of Single-Well Potential Stochastic Resonance Algorithm Aimed at Simultaneous Determination of Multiple Weak Chromatographic Peaks

**DOI:** 10.1155/2014/767018

**Published:** 2014-01-12

**Authors:** Haishan Deng, Shaofei Xie, Bingren Xiang, Ying Zhan, Wei Li, Xiaohua Li, Caiyun Jiang, Xiaohong Wu, Dan Liu

**Affiliations:** ^1^Department of Pharmacy, College of Pharmacy, Nanjing University of Chinese Medicine, No. 138 Xianlin Avenue, Nanjing 210023, China; ^2^Nanjing Changao Pharmaceutical Technology Limited, No. 1 Hengfei Road, Economic and Technological Development Zone, Nanjing 210038, China; ^3^Center for Instrumental Analysis, China Pharmaceutical University, No. 24 Tongjiaxiang, Nanjing 210009, China; ^4^Zhongda Hospital Affiliated to Southeast University, Nanjing 210009, China; ^5^Department of Engineering and Technology, Jiangsu Institute of Economic and Trade Technology, Nanjing 210007, China

## Abstract

Simultaneous determination of multiple weak chromatographic peaks via stochastic resonance algorithm attracts much attention in recent years. However, the optimization of the parameters is complicated and time consuming, although the single-well potential stochastic resonance algorithm (SSRA) has already reduced the number of parameters to only one and simplified the process significantly. Even worse, it is often difficult to keep amplified peaks with beautiful peak shape. Therefore, multiobjective genetic algorithm was employed to optimize the parameter of SSRA for multiple optimization objectives (i.e., *S/N* and peak shape) and multiple chromatographic peaks. The applicability of the proposed method was evaluated with an experimental data set of Sudan dyes, and the results showed an excellent quantitative relationship between different concentrations and responses.

## 1. Introduction

Stochastic resonance algorithm (SRA) [1] is established based on a counterintuitive phenomenon that the signal-to-noise ratio (*S*/*N*) of a weak signal can be amplified significantly in a nonlinear system by making the best of noise instead of filtering it [2]. The algorithm presents the unique advantages for superior detection of useful signal that submerged in heavy noise and provides an entirely new way for the detection of weak chromatographic peaks [3]. It has been successfully applied to many different fields of analytical chemistry, such as pharmaceutical analysis [4], food analysis, [5] and environmental analysis [6].

A nonlinear system is one of the necessary elements of SRA, and the one that is most frequently employed is a bistable system described as double-well potential with two system parameters. The optimization of the system parameters is essential for the application of SRA. The initial goal of the optimization is to pursue the maximal signal-to-noise ratio (*S*/*N*) of the output peak, and, usually, the process of optimization is to search the optimal value one by one within a given range [7]. However, the ill-looking peak shape often annoys the researchers. Therefore, some improved algorithms with two or more parameters were developed to give attention to both the *S*/*N* and the chromatographic peak shape [8, 9]; of course, the workload of parameter optimization inevitably increased. In order to simplify the parameter optimization, Zhang and Xiang developed a single-well potential stochastic resonance algorithm (SSRA) with only one parameter [10]. The algorithm works well in the application of quantitative determination of single weak chromatographic peak [11].

In recent years, simultaneous determination of multiple weak chromatographic peaks via SRA attracts much attention [12, 13], in which the optimization of the parameters becomes even more complicated and time consuming [14]. Genetic algorithms (GAs), which are inspired by the evolutionary principle of “survival of the fittest,” have been proposed as powerful search strategies for tackling complex optimization problems with high efficiencies and robustness [15, 16]. It is desirable that GAs should be suitable for automatically and rapidly optimizing the system parameters of SRA. Wang et al. developed an adaptive single-well stochastic resonance algorithm by applying GA, but only one chromatographic peak of clenbuterol and a single optimization objective (i.e., *S*/*N*) were concerned [17]. In this paper, a multiobjective GA [18] was coupled to SSRA to optimize the system parameters for multiple optimization objectives (i.e., *S*/*N* and peak shape) and multiple chromatographic peaks, where single-well potential was adopted to simplify the theoretical formalism of the algorithm. The applicability of the proposed method was evaluated with an experimental data set of Sudan dyes.

## 2. Theory and Algorithm

### 2.1. Single-Well Potential Stochastic Resonance Algorithm

A simplified nonlinear Langevin equation is employed in the algorithm of stochastic resonance [10, 19] as follows:

(1) $$\begin{array}{c} \frac{dx}{dt}=-U^{\prime }\left(x\right)+I\left(t\right), \end{array}$$

where *I*(*t*) denotes an input signal embedded in a noisy environment, expressed as *I*(*t*) = *S*(*t*) + *N*(*t*). *S*(*t*) is the pure or real signal, and *N*(*t*) is the intrinsic noise generated by instrument. *U*(*x*) is a potential function of the nonlinear system, and that used in SSRA can be expressed by the following equation:

(2) $$\begin{array}{c} U\left(x\right)=-a+\frac{1}{2}bx^{2}. \end{array}$$

**Figure 1:**
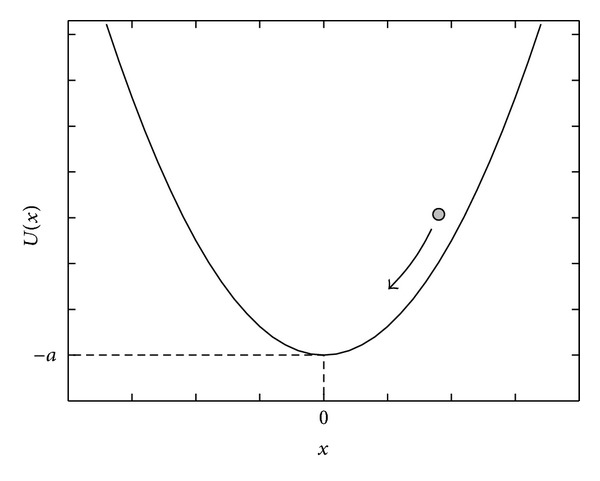
The profile of the single-well potential function.

The profile of *U*(*x*) is shown in Figure 1. As the physical model of SR, the physical meaning of the equations can be explained by the motion of a Brownian particle in the single-well potential. The input signal *I*(*t*) can be viewed as fluctuating force that act upon the particle. The force drives the particle to move along the brim of the single-well potential, and the displacement of the particle, *x*, forms the output signal of the system. In other words, the output chromatogram of the algorithm can be considered as the trajectory of the particle. According to (2), only the parameter *b* affects the profile of the potential well; the parameter *a* decides the vertical position of the well and will be eliminated in the first-order derivative of *U*(*x*) (see (1)). When the parameter *b* takes an appropriate value, the noise may cooperate with the signal properly and the signal will extract energy from the noise. As a result, in the output, the strength of the signal will be increased while that of the noise will be decreased, and the output signal could be driven to a height that cannot be reached via the pure input signal. Finally, a greater *S*/*N* could obtain from the output signal than from the input one.

The analytic solution of Langevin equation is not available, and one has to use a numerical method to approximate it [20]. In this work, a fourth-order Runge-Kutta method was used to obtain the discrete solution [21].

### 2.2. Multiobjective Genetic Algorithm

Genetic algorithms (GAs) are a particular class of evolutionary algorithms that move from one population of “chromosomes” to a new population by using a kind of natural selection together with the genetic inspired operators of crossover, mutation, and so on [22]. A selection operator chooses “chromosomes” in the population that will be allowed to “reproduce.” On average, the fitter “chromosomes” produce more “offspring” than the less fit ones. GAs can search for many noninferior solutions in parallel by maintaining a population of solutions. Therefore, GAs are very suitable for solving the problems of multiobjective optimization [23].

**Figure 2:**
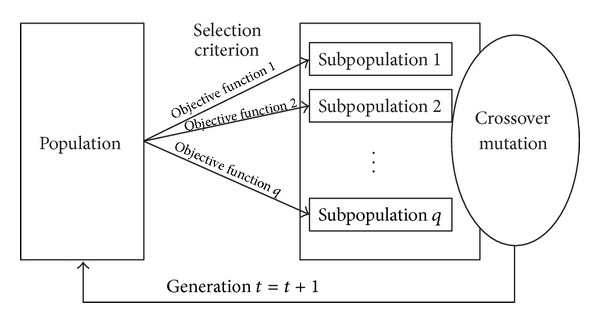
The flowchart of VEGA.

Vector evaluated genetic algorithm (VEGA) is one of the multiobjective GAs that was proposed by Schaffer [24]. In VEGA, a number of subpopulations are generated by performing proportional selection according to each objective function *z*_*i*_ in turn [25]. Assuming the population size of the current generation is *N*, and the number of objectives is *q*, then *q* subpopulations would be generated with the size of *N*/*q* each. A new generation is then obtained by shuffling these subpopulations together, and the operation of crossover and mutation is applied. The flowchart of VEGA is shown in Figure 2.

### 2.3. The Implementation of the Algorithm

As shown in Section 2.1, the parameter *b* is very important for SSRA. In order to get the satisfactory output for each peak, multiobjective genetic algorithm was employed to perform the multiobjective optimization for the parameter *b*. The objective function is defined as follows:

(3) min f1(b)=−∑i=1nSNRi(b)SNRimaxmin f2(b)=∑i=1nSYNi(b)SYNimaxsubject  to 0<b≤1,

where *n* is the number of peaks in the chromatogram. The function consists of two terms: SNR and SYN, which denote the signal-to-noise ratio and the degree of symmetry of the output peaks, respectively. SNR_*i*_(*b*) in (3) is defined as the ratio of the standard deviation of the signal range of the *i*th peak to that of baseline range; that is,

(4) $$\begin{array}{c} {\text{SNR}}_{i}\left(b\right)=\frac{{\text{SD}}_{i}^{\text{peak}}\left(b\right)}{{\text{SD}}_{i}^{\text{noise}}\left(b\right)}. \end{array}$$

SYN_*i*_(*b*) is defined as the ratio of the peak area of the left side to that of the right side of the *i*th peak; that is,

(5) $$\begin{array}{c} {\text{SYN}}_{i}\left(b\right)=\left|\frac{{\text{LA}}_{i}\left(b\right)}{{\text{RA}}_{i}\left(b\right)}-1\right|. \end{array}$$

SNR_*i*_^max^ and SYN_*i*_^max^ are the maximal values of SNR and SYN of the *i*th peak at different parameter *b*, respectively.

The procedures of optimization are as follows.

**Step 1**

Start with a random initial population *P*_*t*_. Set *t* = 0.

**Step 2**

If the ending criterion is satisfied, return *P*_*t*_.

**Step 3**

Randomly sort population *P*_*t*_.

**Step 4**

For each objective *k* (*k* = 1,2,…, *q*), perform the following steps.

*Step  4.1*.  For *i* = 1 + (*k* − 1)*N*_*s*_,…, *kN*_*s*_ (*N*_*s*_ denotes the subpopulation size, *N*_*s*_ = *N*/*q*), assign fitness value *f*(*x*_*i*_) = *z*_*k*_(*x*_*i*_) to the *i*th solution in the sorted population.

*Step  4.2*.  Based on the fitness values assigned in Step  4.1, select *N*_*s*_ solutions between the (1 + (*k* − 1)*N*_*s*_)th and (*kN*_*s*_)th solutions of the sorted population to create subpopulation *P*_*k*_.

**Step 5**

Combine all subpopulations *P*_1_, *P*_2_,…, *P*_*k*_ and apply crossover and mutation on the combined population to create *P*_*t*+1_ of size *N*. Set *t* = *t* + 1, and then go to Step 2.

The algorithm was implemented in Matlab 7 (Mathworks, Natick, MA, USA) by the authors, and the calculations were carried out on an IBM-PC compatible computer (Intel Core Duo CPU 1.83 GHz, memory 1 GB).

## 3. Experimental

### 3.1. Reagents and Standards

All of the Sudan dyes (lot number 40517) and Para Red (lot number 50506) were purchased from Dr. Ehrenstorfer GmbH (Augsburg, Germany), and their chemical purities were 97.5% (Sudan I), 90.0% (Sudan II), 97.0% (Sudan III), 91.0% (Sudan IV), and 95.5% (Para Red), respectively. HPLC grade acetonitrile was purchased from Merck Company (Darmstadt, Germany). Acetic acid (analytical grade) was purchased from Guangdong Xilong Chemical Co., Ltd. (Shantou, Guangdong Province, China). Distilled water was used throughout the study.

Stock solutions (Para Red: 0.515 mg mL^−1^; Sudan I: 0.732 mg mL^−1^; Sudan II: 0.697 mg mL^−1^; Sudan III: 0.823 mg mL^−1^; Sudan IV: 0.721 mg mL^−1^) were prepared in acetonitrile, and working solutions of a series concentration were prepared by diluting the appropriate volumes of the primary stock solutions in acetonitrile. All of the solutions were stored at 4°C.

### 3.2. Chromatographic Conditions

The chromatographic system was the Shimadzu (Tokyo, Japan) LC-10AT vp HPLC system equipped with an LC-10AT vp pump, a 7725 manual injector, and an SPD-10A vp UV-VIS detector. An N2000 chromatography data system (Zhejiang University Star Instrument Technology Co., China) was used, at a sampling frequency of 10 Hz. Separation was carried out at room temperature on a reversed-phase Dikma Diamonsil C18 (150 mm × 4.6 mm i.d., 5 *μ*m) column. The mobile phase consisted of acetonitrile/acidified water (165 mL acetic acid plus 1000 mL water) (15 : 85, v/v). The flow rate was 1.0 mL min^−1^ from 0 to 20 min and 2.0 mL min^−1^ from 20 to 25 min. The detection wavelength was 478 nm and the injection volume was 20 *μ*L.

## 4. Results and Discussion

### 4.1. The Optimization of System Parameter *b*

**Figure 3:**
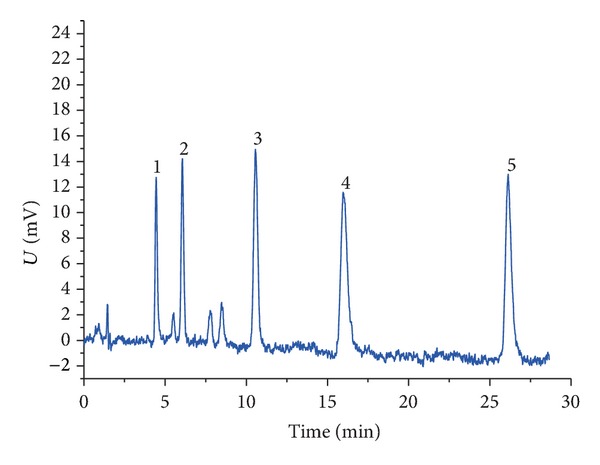
The typical chromatogram of Sudan dyes. (1: Para Red; 2: Sudan I; 3: Sudan II; 4: Sudan III; 5: Sudan IV).

The typical chromatogram of Sudan dyes is presented in Figure 3. The retention times of Para Red and Sudan I~IV were around 4.5, 6.0, 10.4, 15.8, and 25.9 min, respectively. In order to avoid from being affected by the peaks of foreign components, which will also absorb energy from noise, a section of signal during the period of 2 to 29 min was chosen as the input for SSRA.

**Figure 4:**
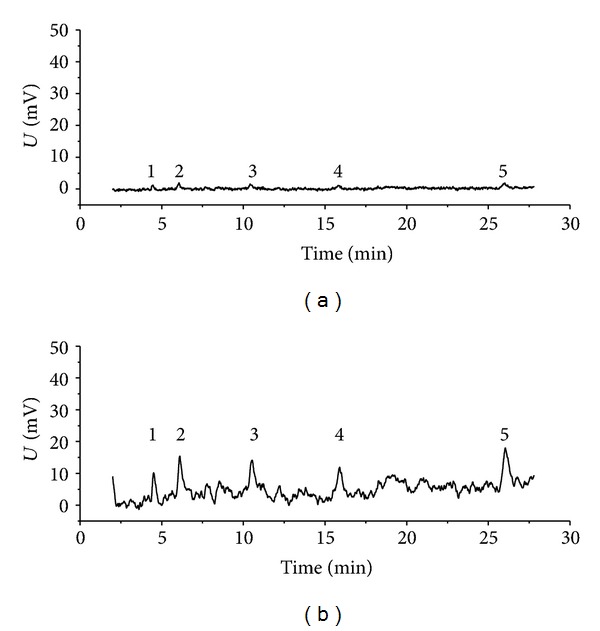
The weak peaks of Sudan dyes were amplified by SSRA with the parameters optimized via multiobjective genetic algorithm. (a) The chromatogram of Sudan dyes solution at extreme low concentrations (1: Para Red, 5.76 ng mL^−1^; 2: Sudan I, 8.16 ng mL^−1^; 3: Sudan II, 20.08 ng mL^−1^; 4: Sudan III, 19.76 ng mL^−1^; 5: Sudan IV, 48.48 ng mL^−1^); (b) the chromatogram obtained via SSRA.

Figure 4(a) shows the chromatogram of Sudan dyes at extreme low concentrations; the peaks were too weak to meet the requirement of analysis. Therefore, SSRA was employed to enhance the peaks, where the parameter *b* was optimized by multiobjective genetic algorithm (VEGA). The parameters that were used in VEGA are as follows:

1. number of individuals: 100;
2. maximum number of generations: 100;
3. precision of variables: 20;
4. generation gap: 0.9.

The objective function reached the minimum at the 49th iteration, and the satisfied value of the parameter *b*  (*b* = 0.0171) is obtained.  Figure 4(b) illustrates how the peaks were enhanced and the chromatogram was improved by SSRA.

### 4.2. Quantitative Analysis of Sudan I~IV

**Figure 5:**
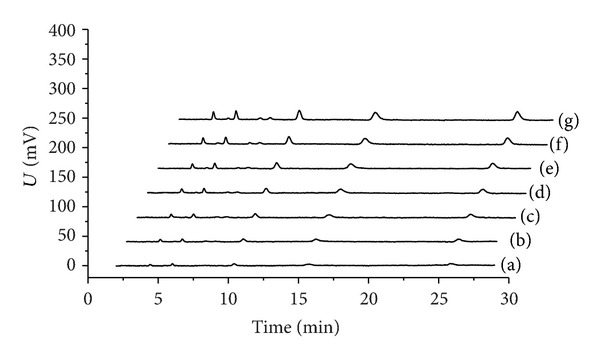
The original chromatograms from Para Red (1), Sudan I (2), Sudan II (3), Sudan III (4), and Sudan IV (5). (a) 14.4, 20.4, 50.2, 49.4, and 121.2 ng mL^−1^; (b) 21.6, 30.6, 75.3, 74.1, and 181.8 ng mL^−1^; (c) 28.8, 40.8, 100.4, 98.8, and 242.4 ng mL^−1^; (d) 36.0, 51.0, 125.5, 123.5, and 303 ng mL^−1^; (e) 43.2, 61.2, 150.6, 148.2, and 363.6 ng mL^−1^; (f) 57.6, 81.6, 200.8, 197.6, and 484.8 ng mL^−1^; (g) 72.0, 102, 251, 247, and 606 ng mL^−1^.

**Figure 6:**
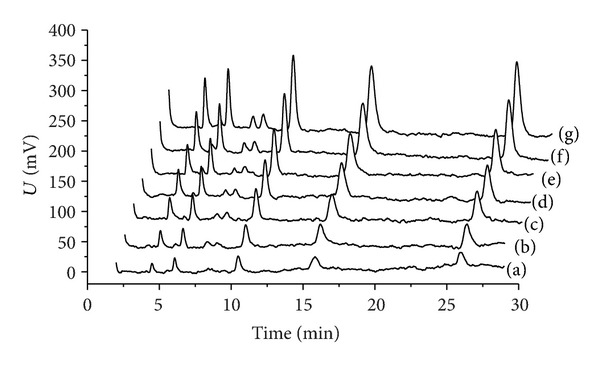
The chromatograms obtained via SSRA.

Figure 5 presents the original chromatograms of Sudan dyes at different concentrations. SSRA was used to amplify the chromatographic peaks present at each concentration. Although all samples have different strengths in different concentrations, the same value of the parameter *b*  (*b* = 0.0171) will be used for them to keep the quantitative relationship of the output signals.  Figure 6 shows how the peaks of all the 5 components at different concentrations can be processed and improved by SSRA; the peaks were amplified obviously and the shape of the peaks was satisfactory.  Table 1 lists the chromatographic areas of Sudan dyes at different concentrations obtained using SSRA; the linearity was excellent over the experimental concentration ranges.

## 5. Conclusion

Multiobjective genetic algorithm is competent for the multiobjective parameter optimization of SSRA. It not only endow SSRA with the ability of detecting multiple weak chromatographic peaks simultaneously, but also can give attention to both the *S*/*N* and peak shape; the process of the parameter optimization is easy and rapid. It can be expected that SSRA should become a promising tool for multicomponent trace analysis with the help of multiobjective genetic algorithm.

## Figures and Tables

**Table 1:** Calibration curves of Sudan dyes obtained via SSRA.

	a	b	c	d	e	f	g	Linear regression curve
Para Red								
Conc.^1^	14.4	21.6	28.8	36	43.2	57.6	72	*A* = (156.1 ± 6.999)*c* + (1231 ± 303.6) *r* = 0.9950, SD = 348.1
*A* ^2^	2916.6	4691.8	6107.6	7119.1	8108.6	10087.2	12301.7	
Sudan I								
Conc.	20.4	30.6	40.8	51	61.2	81.6	102	*A* = (125.5 ± 3.575)*c* + (1599 ± 219.6) *r* = 0.9980, SD = 251.9
*A*	4340.9	5189.6	7012.2	7959.4	8931.8	11877.7	14510	
Sudan II								
Conc.	50.2	75.3	100.4	125.5	150.6	200.8	251	*A* = (96.20 ± 2.178)*c* + (3150 ± 329.2) *r* = 0.9987, SD = 377.6
*A*	8201	10266	12857	14852.2	18052.2	21973.5	27599.5	
Sudan III								
Conc.	49.4	74.1	98.8	123.5	148.2	197.6	247	*A* = (161.1 ± 5.481)*c* − (307.1 ± 815.5) *r* = 0.9971, SD = 935.2
*A*	7171.1	11539.4	16825.9	18473.6	24582.9	30915.7	39575.2	
Sudan IV								
Conc.	121.2	181.8	242.4	303	363.6	484.8	606	*A* = (61.77 ± 2.273)*c* + (1171 ± 829.8) *r* = 0.9966, SD = 951.6
*A*	7367.9	12883.9	16985.5	20513.8	23914.1	29937.4	38845.5	

^1^Conc.: concentration; ^2^ *A*: peak area obtained via SSRA.
